# Supplementary material for: Emergence and spread of two SARS-CoV-2 variants of interest in Nigeria
Source: Nat Commun. 2023 Feb 13;14:811. doi: 10.1038/s41467-023-36449-5 (PMC9924892; doi:10.1038/s41467-023-36449-5)
Supplement: Supplementary file 1 — Supplementary Information [file 41467_2023_36449_MOESM1_ESM.pdf]

Supplementary material

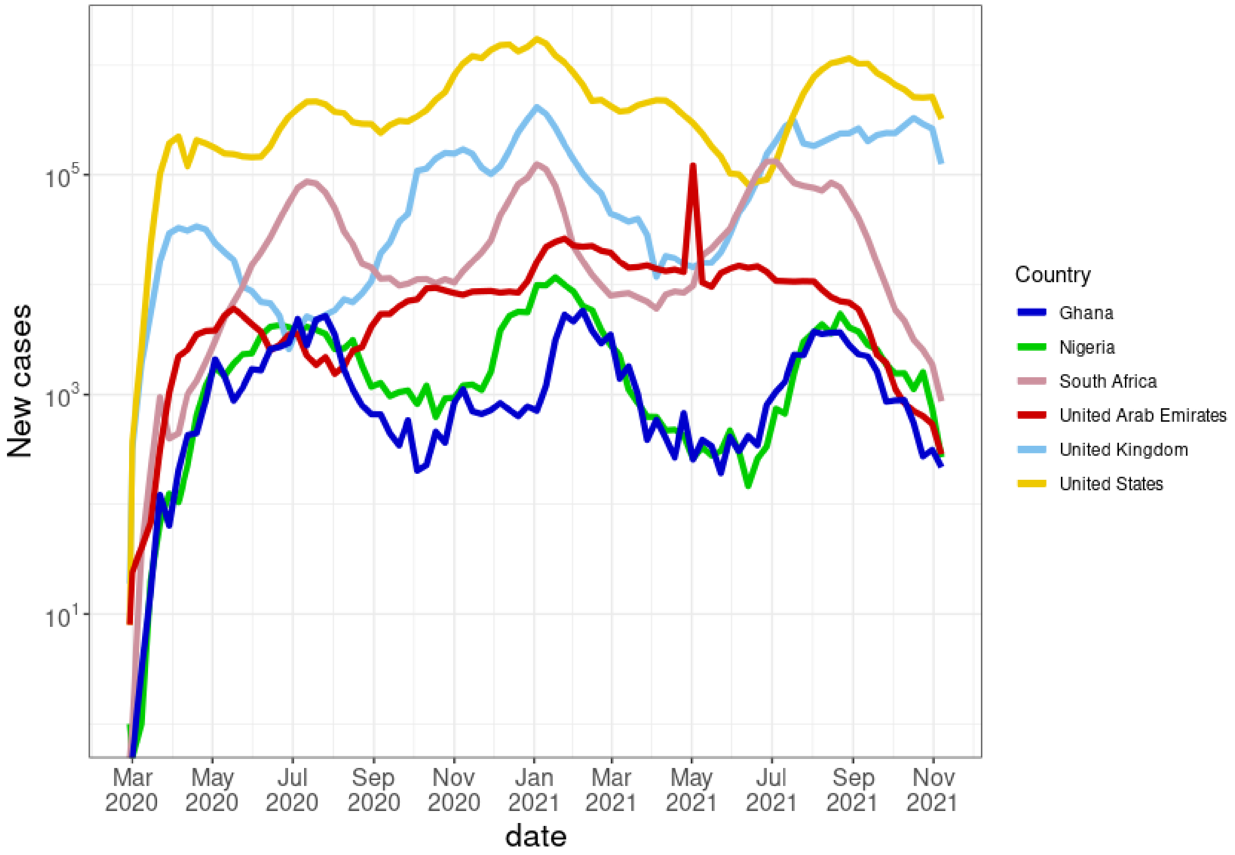

SFigure 1: COVID-19 new cases per week as seen in Nigeria and countries with highest flight traffic in and out of Nigeria from the four major connected regions with the y-axis transformed to a log scale.

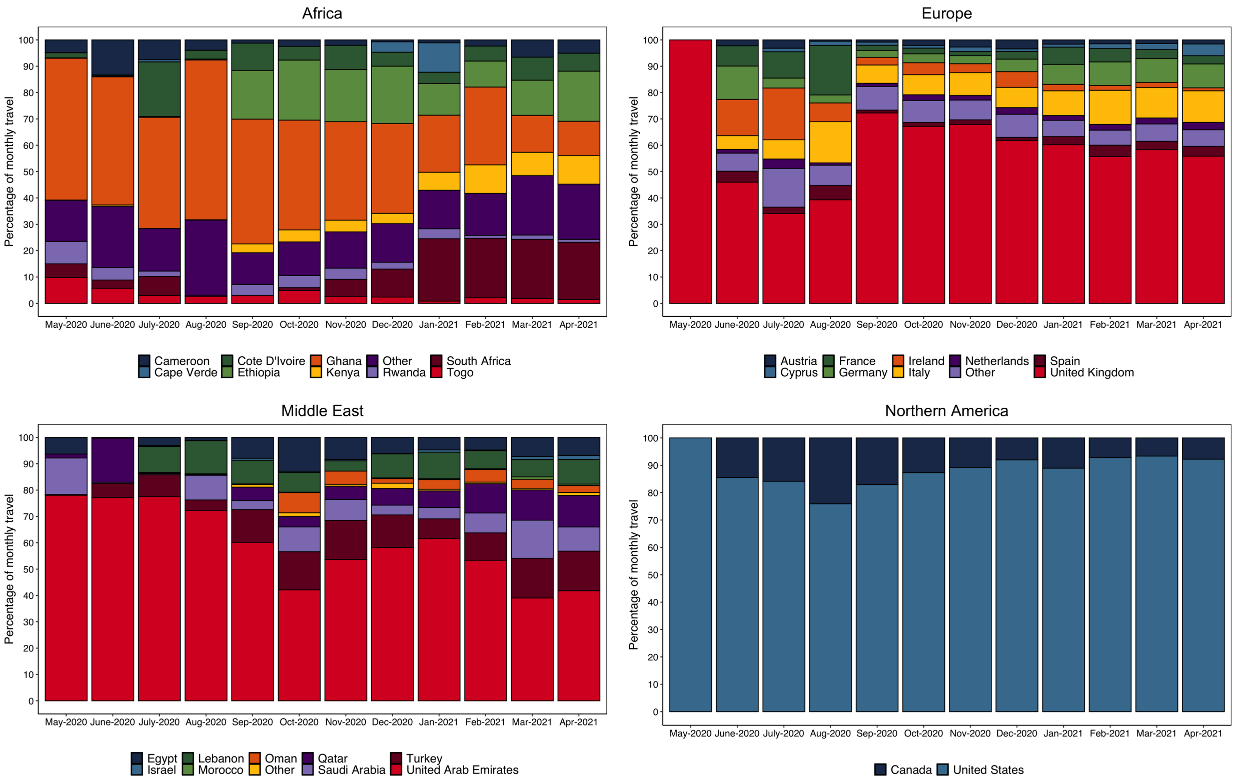

SFigure 2: Percentage of incoming travel from the four major connected regions by country as per Figure 2

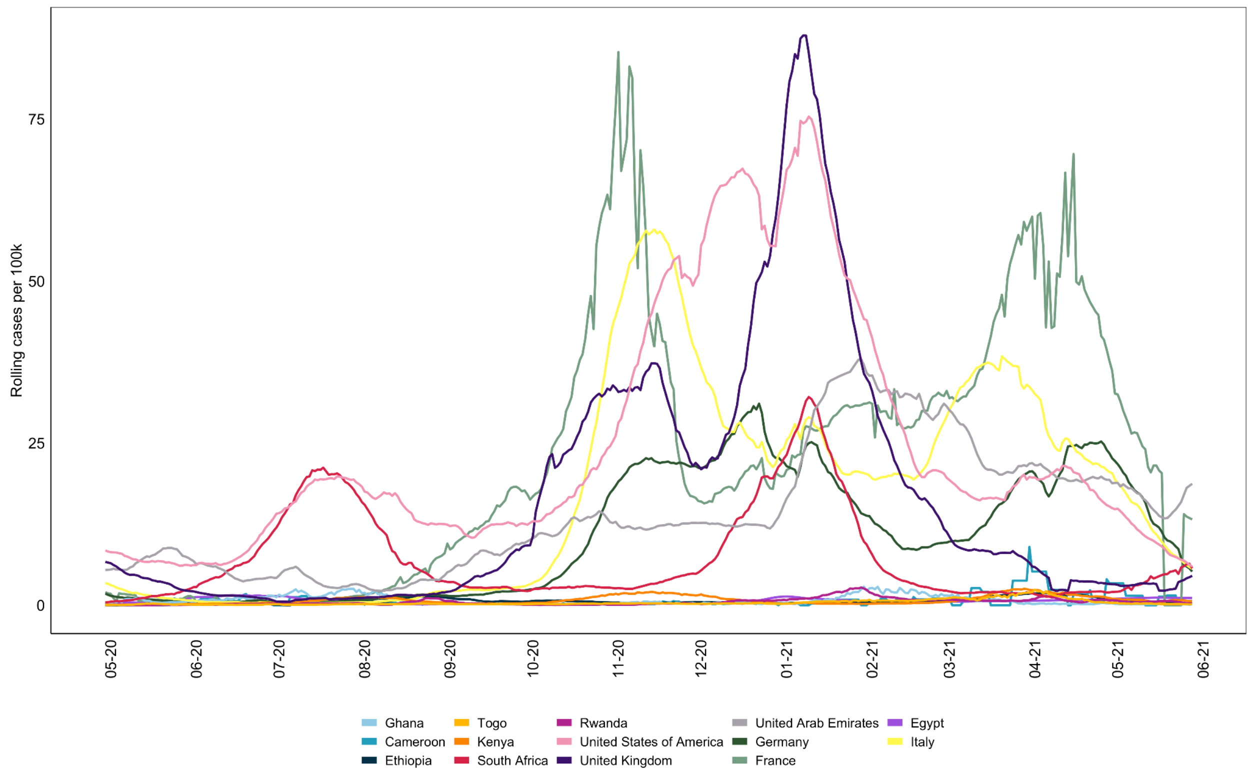

SFigure 3: Population adjusted rolling average of new daily cases for countries with the highest incoming air travel as per Figure S2.

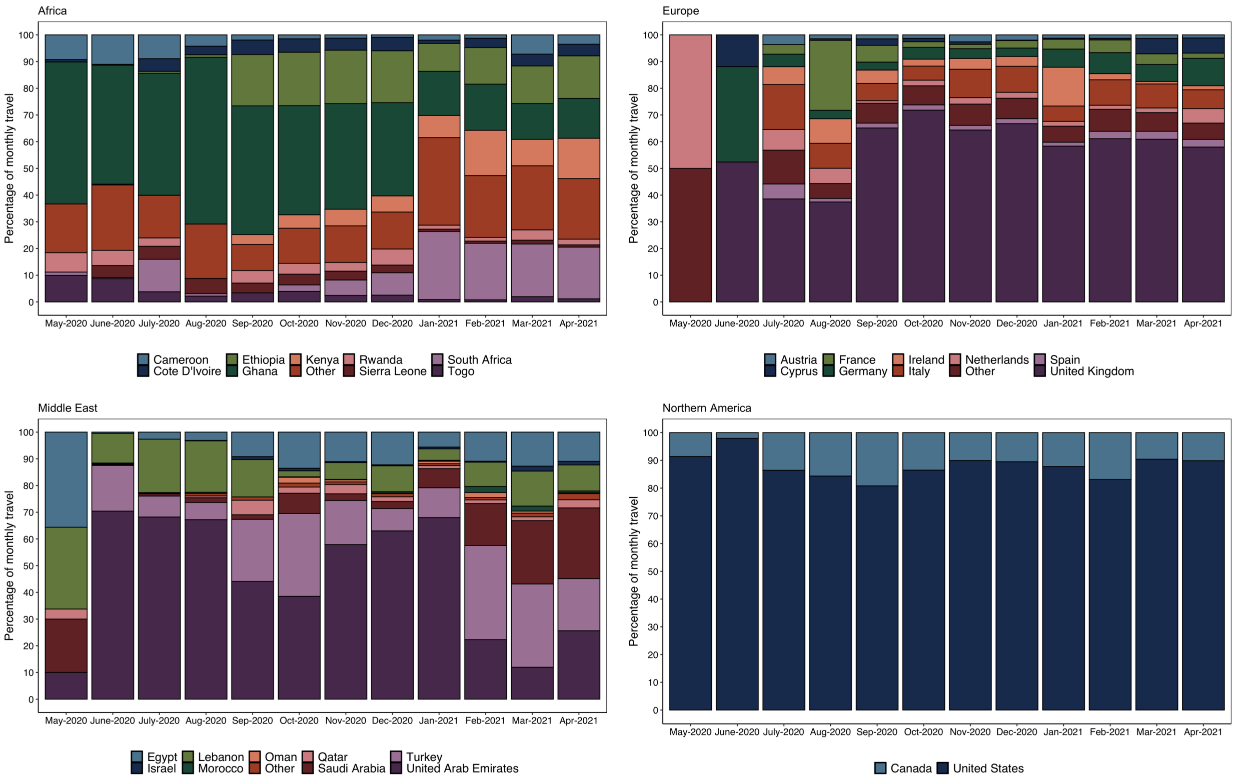

SFigure 4: Percentage of outgoing travel to the four major connected regions by country as per Figure 3

Data Availability

GISAID Identifier: EPI_SET_221227pc
doi: https://doi.org/10.55876/gis8.221227pc
All genome sequences and associated metadata in this dataset are published in GISAID's EpiCoV database. To view the contributors of each individual sequence with details such as accession number, Virus name, Collection date, Originating Lab and Submitting Lab and the list of Authors, visit 10.55876/gis8.221227pc

Data Snapshot

- EPI_SET_221227pc is composed of 3,959 individual genome sequences.
- The collection dates range from 2020-05-14 to 2021-09-28;
- Data were collected in 68 countries and territories;
- All sequences in this dataset are compared relative to hCoV-19/Wuhan/WIV04/2019 (WIV04), the official reference sequence employed by GISAID (EPI_ISL_402124). Learn more at https://gisaid.org/WIV04.

GISAID Identifier: EPI_SET_221227vp
doi: https://10.55876/gis8.221227vp
All genome sequences and associated metadata in this dataset are published in GISAID's EpiCoV database. To view the contributors of each individual sequence with details such as accession number, Virus name, Collection date, Originating Lab and Submitting Lab and the list of Authors, visit 10.55876/gis8.221227vp

Data Snapshot

- EPI_SET_221227vp is composed of 8,415 individual genome sequences.
- The collection dates range from 2020-03-25 to 2021-09-23;
- Data were collected in 91 countries and territories;
- All sequences in this dataset are compared relative to hCoV-19/Wuhan/WIV04/2019 (WIV04), the official reference sequence employed by GISAID (EPI_ISL_402124). Learn more at https://gisaid.org/WIV04.
