# Supplementary material for: Emergence and spread of two SARS-CoV-2 variants of interest in Nigeria
Source: Nat Commun. 2023 Feb 13;14:811. doi: 10.1038/s41467-023-36449-5 (PMC9924892; doi:10.1038/s41467-023-36449-5)
Supplement: Supplementary file 3 — Reporting Summary [file 41467_2023_36449_MOESM3_ESM.pdf]

nature portfolio

Corresponding author(s): Prof. Christian Happi

Last updated by author(s): Jan 11, 2023

Reporting Summary

Nature Portfolio wishes to improve the reproducibility of the work that we publish. This form provides structure for consistency and transparency in reporting. For further information on Nature Portfolio policies, see our Editorial Policies and the Editorial Policy Checklist.

Statistics

For all statistical analyses, confirm that the following items are present in the figure legend, table legend, main text, or Methods section.

n/a	Confirmed	
☐	☒	The exact sample size (*n*) for each experimental group/condition, given as a discrete number and unit of measurement
☒	☐	A statement on whether measurements were taken from distinct samples or whether the same sample was measured repeatedly
☒	☐	The statistical test(s) used AND whether they are one- or two-sided<br>*Only common tests should be described solely by name; describe more complex techniques in the Methods section.*
☒	☐	A description of all covariates tested
☐	☒	A description of any assumptions or corrections, such as tests of normality and adjustment for multiple comparisons
☒	☐	A full description of the statistical parameters including central tendency (e.g. means) or other basic estimates (e.g. regression coefficient) AND variation (e.g. standard deviation) or associated estimates of uncertainty (e.g. confidence intervals)
☒	☐	For null hypothesis testing, the test statistic (e.g. *F*, *t*, *r*) with confidence intervals, effect sizes, degrees of freedom and *P* value noted<br>*Give P values as exact values whenever suitable.*
☐	☒	For Bayesian analysis, information on the choice of priors and Markov chain Monte Carlo settings
☒	☐	For hierarchical and complex designs, identification of the appropriate level for tests and full reporting of outcomes
☒	☐	Estimates of effect sizes (e.g. Cohen's *d*, Pearson's *r*), indicating how they were calculated

Our web collection on statistics for biologists contains articles on many of the points above.

Software and code

Policy information about availability of computer code

Data collection	No software was used for data collection
Data analysis	Genome assembly was performed with viral-ngs pipeline v2.1.19 (https://github.com/broadinstitute/viral-ngs). PANGOLIN v3.1.12 and NextClade v1.3.0 was used for lineage and clade assignment. MAFFT v7.490, IQTREE v2.1.2, Treetime v0.92, and Nextstrain v3.0.3 were used for multiple sequence alignment and phylogenetic analyses. We used BEAST v1.10.5, Tracer v1.7, TreeAnnotator v1.10 to conduct Bayesian time-scaled phylogenies and discrete trait analysis.

For manuscripts utilizing custom algorithms or software that are central to the research but not yet described in published literature, software must be made available to editors and reviewers. We strongly encourage code deposition in a community repository (e.g. GitHub). See the Nature Portfolio guidelines for submitting code & software for further information.

Data

Policy information about availability of data

All manuscripts must include a data availability statement. This statement should provide the following information, where applicable:

- Accession codes, unique identifiers, or web links for publicly available datasets
- A description of any restrictions on data availability
- For clinical datasets or third party data, please ensure that the statement adheres to our policy

The datasets and associated metadata used in this study are available in GISAID's EpiCoV database under the EPI_SET_ID accession numbers EPI_SET_221227vp and EPI_SET_221227pc. XML files used for BEAST analysis are deposited in GitHub: https://github.com/acegid/SARS-CoV-2_Manuscript_Supplemental_Data. All

SARS-CoV-2 consensus genome assemblies generated in this study are deposited in NCBI GenBank under the accession numbers: OQ050230 to OQ052977. DNA sequences have been deposited in NCBI SRA under the BioProject PRJNA916503. Source data are provided as a Source Data file. Reference genome Wuhan-Hu-1 available at GenBank under accession MN908947.3. Air travel data can be requested for release from Bluedot (info@bluedot.global), with use pending approval by Bluedot

Human research participants

Policy information about studies involving human research participants and Sex and Gender in Research.

Reporting on sex and gender	Sex and gender information were not reported in this study
Population characteristics	Samples were collected from both male and female population between all ages
Recruitment	Participant recruitment was voluntarily based on individuals who consented to have their samples collected and used for this study. Written informed consent was obtained directly from the patient as part of the routine surveillance program in Nigeria. Samples were collected from people who reported to community testing centres for COVID-19 tests (travellers included) and hospitalised individuals from February 2020 to October 2021 across the country. There were no self-selection biases during the study design and participant enrollment that could have impacted the outcome of findings.
Ethics oversight	National Health Research Ethics Committee of Nigeria (NHREC)

Note that full information on the approval of the study protocol must also be provided in the manuscript.

Field-specific reporting

Please select the one below that is the best fit for your research. If you are not sure, read the appropriate sections before making your selection.

[x] Life sciences [ ] Behavioural & social sciences [ ] Ecological, evolutionary & environmental sciences

For a reference copy of the document with all sections, see nature.com/documents/nr-reporting-summary-flat.pdf

Life sciences study design

All studies must disclose on these points even when the disclosure is negative.

Sample size	No sample size calculations were made. Samples were randomly selected from PCR positive SARS-CoV-2 samples for whole genome sequencing. These were from 26 out of 37 states including the Federal Capital Territory in Nigeria. This sample size was deemed sufficient as it was more than 2.5% of total confirmed cases from all geopolitical zones in Nigeria.
Data exclusions	Samples with high CT values (> 30) were excluded for whole genome sequencing. Additionally, consensus genomes that have more than 5% ambiguity in their genomes were excluded for phylogenetics construction
Replication	The datasets were re-analyzed using the same version of software on a different machine at a different institution in order to verify reproducibility. This attempt of replication was successful.
Randomization	Positive samples that passed the inclusion criteria were selected through simple random selection to ensure that every sample has equal probability of being selected. For samples from states/regions with small sample size, all samples were analyzed, however, locations with large sample sizes were downsampled and normalized with a random selection to match the other groups.
Blinding	The researchers were blinded to sample selection and group allocation during data analysis as every sample were stripped of identifiers to ensure protection of participants and also non-bias during sample collection

Reporting for specific materials, systems and methods

We require information from authors about some types of materials, experimental systems and methods used in many studies. Here, indicate whether each material, system or method listed is relevant to your study. If you are not sure if a list item applies to your research, read the appropriate section before selecting a response.

Materials & experimental systems

n/a	Involved in the study
☒	☐ Antibodies
☒	☐ Eukaryotic cell lines
☒	☐ Palaeontology and archaeology
☒	☐ Animals and other organisms
☒	☐ Clinical data
☒	☐ Dual use research of concern

Methods

n/a	Involved in the study
☒	☐ ChIP-seq
☒	☐ Flow cytometry
☒	☐ MRI-based neuroimaging
